# Students perspective on attendance monitoring in undergraduate obstetrics and gynecology

**DOI:** 10.2147/AMEP.S131475

**Published:** 2017-02-20

**Authors:** Prashant Bamania, Nicholas J Burstow

**Affiliations:** Faculty of Medicine, Imperial College London, London, Kensington, UK

**Dear editor**

We read with great interest the article by Deane and Murphy^1^ regarding student and staff experiences of attendance monitoring in undergraduate obstetrics and gynecology (OBG). We agree with the findings of this cross-sectional survey, which show that attendance monitoring was acceptable to both staff and students.^1^ Student attendance is considered a vital aspect to professional development. Furthermore, studies have demonstrated a positive correlation between student attendance and academic performance in both clinical- and tutorial-based learning environments.^2^

As fifth-year medical students having just finished our OBG placement at Imperial College London, we were also subjected to attendance monitoring and the use of logbooks. Deane and Murphy^1^ found that, although students found monitoring attendance to be acceptable, attendance was only of value at “educationally beneficial” activities. Our experiences during our OBG placement were similar, with most students attending when they felt their attendance would benefit their learning.

Although monitoring attendance may contribute to a student’s personal and professional development, it raises practicality questions for both students and staff. This study used a paper-based system of monitoring attendance, whereby students obtained staff signatures for each activity attended in a paper logbook. Despite this extra administrative burden, the study found that both students and staff “did not regard completing logbooks as an excessive burden”. We also found this to be the case from our experiences with logbooks during our OBG placement – students rarely encounter issues with obtaining signatures from staff members.

One important difference to note is how Imperial College London uses logbooks to monitor our attendance. Rather than requiring a signature for each attendance, as in this study, we are required to record our learning experiences from each clinical experience in our logbook. Our lead consultant for the placement then reads over these entries twice (once midway through and again at the end of the placement), and “signs us off” if we have demonstrated suitable attendance.

Another difference between this study and our experiences is the format of the logbook. Whereas this study used paper logbooks, Imperial College London uses electronic logbooks via an iPad given to each student by the University. Our logbooks also contain sections for notes and student reflections, as well as sign-off forms for clinical competency skills. In addition, there is also space where staff may give detailed written feedback of how the student performed in their patient interaction, describing any strengths or weaknesses. Although a more time-consuming process, we found that most members of staff are willing to give us constructive written feedback in the logbook, furthering the educational benefit of our attendance. We are able to easily keep track of our attendances and competencies by combining all these things into one, readily transportable electronic logbook.

With regard to logbooks, studies have raised issues over the accuracy of the recorded information without the need for signatures.^3^,^4^ The logbook used in this study required a signature for each recorded experience. However, it found that some students forgot their logbook, or forgot to obtain a signature on the day, leading to retrospective “signings off”, with no way for the member of staff to verify whether the student had actually attended. To address these issues, we suggest the use of an electronic logbook whereby multiple experiences can be recorded in the students’ own time, with only one signature required at the end of the placement as previously described.

The requirement for signatures and thorough feedback required by Imperial meant that we attended all timetabled sessions, and in doing so found interactions with health care staff to be more productive. Over the course of our placement, we felt our patient communication skills improved. This study reported similar findings, with students having to “push themselves verbally to interact with clinical staff”, facilitating greater access for learning opportunities.

In conclusion, we acknowledge the importance of monitoring students’ attendance in the clinical environment, and the way in which the study authors attempted to do so, adding to the relatively sparse literature on the subject. As an alternative to “pen and paper”, we suggest the use of electronic systems in attendance monitoring in the clinical environment. Monitoring attendance encourages students to attend their clinical placements, contributing to their personal and professional development and ultimately ensuring they fulfill the criteria required by the General Medical Council to be a competent doctor.^5^
